# WIDENING SEXUAL ORIENTATION INEQUITIES IN SMOKING AMONG OLDER ADULTS IN THE UNITED STATES, 2015–2019

**DOI:** 10.1093/geroni/igad104.2575

**Published:** 2023-12-21

**Authors:** Jie Yang

**Affiliations:** East Carolina University, Greenville, North Carolina, United States

## Abstract

A recent editorial in Tobacco Control noted the neglect of older adults in the field of tobacco control, arguing that we must do better. This study seeks to answer that call and amplify its message by assessing what is missed in health equity research when older adults are ignored in another area of inequity: differences in smoking by sexual orientation. We used pooled data from the 2015-2019 National Survey on Drug Use and Health. We estimated smoking prevalence for older adults aged ≥50 (N=43,956) by gender and sexual identity. Following the National Center for Health Statistics guidelines for analysis of trends, we used logistic regression to examine the association between continuous year (time) and smoking controlling for sexual identity. We also tested inequities in change using an interaction between year and sexual identities. In Model 1, there was a significant decreasing trend of smoking among all older adults: With each passing year, the odds of smoking went down (aOR 0.98, 95%CI 0.95-1.00). Heterosexual men, gay men, bisexual women), and lesbian women had a higher likelihood of smoking than heterosexual women. In Model 2, there was a significant interaction between year and sexual identity (aOR 1.34, 95%CI 1.02-1.75). That is, the change in likelihood of smoking from 2015 to 2019 for bisexual women was significantly different than the change for heterosexual women. Widening inequities in current smoking across some sexual identities among older adults indicate the importance of ensuring cessation campaigns for LGB adults are not just reaching younger adults.

